# Prenatal exposure to recreational drugs affects global motion perception in preschool children

**DOI:** 10.1038/srep16921

**Published:** 2015-11-19

**Authors:** Arijit Chakraborty, Nicola S. Anstice, Robert J. Jacobs, Linda L. LaGasse, Barry M. Lester, Trecia A. Wouldes, Benjamin Thompson

**Affiliations:** 1 School of Optometry and Vision Science, Faculty of Medical and Health Sciences, University of Auckland, Private Bag 92019, Auckland, 1142, New Zealand; 2 Brown Center for the Study of Children at Risk, Warren Alpert Medical School at Brown University, 101 Dudley Street, Providence, RI 02905, USA; 3 Department of Psychological Medicine, Faculty of Medical and Health Sciences, University of Auckland, Private Bag 92019, Auckland 1142, New Zealand; 4 School of Optometry and Vision Science, Faculty of Science, University of Waterloo, 200 Columbia Street West, Waterloo, Ontario, ON N2L, Canada

## Abstract

Prenatal exposure to recreational drugs impairs motor and cognitive development; however it is currently unknown whether visual brain areas are affected. To address this question, we investigated the effect of prenatal drug exposure on global motion perception, a behavioural measure of processing within the dorsal extrastriate visual cortex that is thought to be particularly vulnerable to abnormal neurodevelopment. Global motion perception was measured in one hundred and forty-five 4.5-year-old children who had been exposed to different combinations of methamphetamine, alcohol, nicotine and marijuana prior to birth and 25 unexposed children. Self-reported drug use by the mothers was verified by meconium analysis. We found that global motion perception was impaired by prenatal exposure to alcohol and improved significantly by exposure to marijuana. Exposure to both drugs prenatally had no effect. Other visual functions such as habitual visual acuity and stereoacuity were not affected by drug exposure. Prenatal exposure to methamphetamine did not influence visual function. Our results demonstrate that prenatal drug exposure can influence a behavioural measure of visual development, but that the effects are dependent on the specific drugs used during pregnancy.

Prenatal exposure to recreational drugs is a growing problem^1^,^2^. Research into the impact of prenatal drug exposure has focused primarily on cognitive and motor development following maternal use of opioids such as cocaine or methadone^3^,^4^,^5^. More recently, the effects of prenatal exposure to amphetamine-type stimulants such as methamphetamine have been investigated because of the high prevalence of users, particularly within Australasia^6^,^7^. Such studies include the Infant Development, Environment and Lifestyle (IDEAL) study which has reported impaired motor development in young children exposed prenatally to methamphetamine^6^,^8^.

The effect of prenatal drug exposure on visual development is less well understood; however current evidence suggests that prenatal drug exposure does affect the visual system. A recent large-scale retrospective comparative case series found substantial increases in the rate of strabismus and nystagmus in infants with prenatal drug exposure that persisted at 5 years of age^9^. There is also recent evidence that prenatal drug exposure can affect visual cortex function. McGlone *et al.* found that 6-month old infants with prenatal exposure to methadone exhibited abnormal visual evoked potentials^10^, suggesting disruption within the visual pathway. Abnormal visual cortex responses have also been reported in a small group of children with prenatal exposure to amphetamine^11^.

Participants in the IDEAL study were recruited to two groups on the basis of prenatal methamphetamine exposure (methamphetamine exposed vs. controls). Many mothers of methamphetamine-exposed children were poly-drug users. The control group included children who were also exposed to a range of drug combinations with the exception of methamphetamine as well as non-drug exposed children. Prenatal drug exposure was verified objectively by meconium analysis. Therefore, this group of children provided a unique opportunity to study the effects on visual development of prenatal exposure to a range of substances.

The aim of this study was to investigate higher-level processing within the extrastriate visual cortex in 4.5-year-old children enrolled in the New Zealand arm of the IDEAL study. Specifically, we assessed the effect of prenatal drug exposure on a psychophysical measure of global motion perception, which is dependant on visual areas such as V5 in the dorsal visual stream. This particular stream connects the magnocellular layers of the lateral geniculate nucleus, the primary visual cortex and extrastriate areas such as V3a and V5 within the occipito-parietal cortex^12^,^13^. Visual areas V3a and V5 process global motion by integrating local motion signals from the primary visual cortex^14^.

Global motion perception was chosen as a measure of extrastriate visual function because it has been suggested that dorsal stream function is particularly vulnerable to the effects of abnormal neurodevelopment; the dorsal stream vulnerability hypothesis^15^,^16^. For example, impaired global motion perception has been found in children born with developmental risk factors such as William’s syndrome, dyslexia, autistic spectrum disorder and foetal alcohol syndrome^17^,^18^,^19^,^20^,^21^,^22^. Poor global motion perception has also been associated with deficits in visuomotor tasks involving reaching, grasping, and locomotor action^17^,^23^,^24^,^25^. Performance of such tasks is mediated by regions of the posterior parietal lobe that receive input from the dorsal visual stream^26^,^27^. In this context, our choice of global motion perception was further motivated by the delayed motor development at birth^28^ and between 1 and 3 years of age^8^ that has been found within the IDEAL study cohort.

## Methods

The Auckland District Health Board ethics committee, the Waitematā District Health Board ethics committee, and the New Zealand Ministry of Health Northern Regional Ethics Committee approved the study. All caregivers provided full informed consent and the study conformed to the principles of the Declaration of Helsinki.

The design of the New Zealand IDEAL study is described in detail by Wouldes *et al.*^6^,^8^. Mothers were referred to the study by independent or hospital based midwives and prenatal drug use was assessed using the Substance Use Inventory^29^. In addition, meconium was collected soon after birth and was sent to the United States Drug Testing Laboratory, Des Plaines, Illinois, USA for drug metabolite analysis using gas chromatography – mass spectrometry in order to objectively determine prenatal drug exposure^6^. All methamphetamine and non-methamphetamine exposed children were matched for socio-economic status, maternal education level (fifth form certificate achieved or not achieved), birth weight (grouped as <1500 g, 1500–2500 g and >2500 g) and gestational age.

The Wechsler Preschool and Primary Scale of Intelligence (WPPSI)-III^30^ was administered by experienced assessors as part of a comprehensive neurodevelopmental assessment conducted at 4.5 years of age for the IDEAL study cohort. The verbal IQ score from the WPPSI-III was used within this study of visual funtion to control for any effects of cognitive or verbal development on performance of the global motion perception task. The Verbal IQ score was chosen as it does not include any measures that rely directly on visual function.

The IDEAL study cohort were assessed at 4.5 years of age because children start school after 5 years of age in New Zealand and therefore data collected at an older age may have been influenced by differential educational experiences. Measures of visual function were included in the 4.5 year assessment protocol because 4.5-year-old children are capable of completing behavioural measures of visual acuity and global motion perception.

### Measurement of global motion perception

Global motion perception was assessed using random dot kinematograms (RDK) that consisted of 100 circular dots (dot diameter 0.24°, dot density 1.27 dot/deg^2^) presented within a circular aperture (10° diameter) at a viewing distance of 60 cm. Dot speed was 6°/second and the presentation time was 1 second. These parameters were chosen on the basis of previous studies that have investigated global motion perception in children^18^,^31^,^32^. Dots had a limited lifetime, whereby each dot had a 5% chance of disappearing on each frame and being redrawn in a random location. Dots were presented at maximum brightness (137 cd/m^2^) on a grey background (45 cd/m^2^) and dot contrast was 0.51 as defined using the Michelson equation: (L_dots_ − L_background_)/(L_dots_ + L_background_). Signal dots moved coherently upwards or downwards and noise dots moved in random directions. Dots that reached the edge of the stimulus aperture were wrapped around to maintain an even dot density. Stimuli were presented on a 15” Dell cathode ray tube (CRT) monitor (model: E771p) with a 120 Hz refresh rate and 1024 × 768 resolution. Stimuli were generated using MATLAB 2013a and psychtoolbox-3^33^. Prior to threshold measurement, children were familiarized with the stimuli and task. First, the children were presented with 100% coherent (all signal dots), high contrast RDKs moving up or down. After 4 successive correct responses at the 100% coherence level, the experimenter varied manually the direction and coherence of the RDK to demonstrate the appearance of RDKs with different coherence levels. Once the child was familiar with the stimulus and task, a 2-down-1-up adaptive staircase test was used to vary the coherence of the RDK to measure a motion coherence threshold^34^. Children were asked to judge whether the dots were moving mostly up or mostly down and could respond verbally and/or by pointing to the top or bottom of the screen. The staircase began at 100% coherence and had a proportional step size of 50% until the first reversal and 25% thereafter. The staircase was terminated after 5 reversals and the threshold was calculated by averaging the last 4 reversals.

A comprehensive vision screening was also conducted to rule out the influence of ocular disease, significant refractive error or other visual deficits on global motion perception. This screening consisted of measuring habitual visual acuity using the crowded Keeler logMAR chart, stereoacuity with the VAO fly stereotest, ocular motility using a cover test, a broad H-test and a 20-prism-diopter base out test. Ocular health was also assessed using the red reflex test, external inspection and pupillary evaluation.

### Statistical analysis

To understand the effect of individual drugs on global motion perception, a univariate general linear model was constructed with fixed factors of drug exposure, sex and ethnicity and covariates of verbal IQ, stereoacuity and better eye habitual visual acuity. The fixed factor of drug exposure coded exposure to each of the following drugs as yes or no; nicotine, alcohol, marijuana and methamphetamine. Note that exposure to opiates was an exclusion criterion for the IDEAL study. Significant main effects and interactions were investigated using post-hoc paired sample t-tests.

As a secondary analysis, we assessed the relationship between the estimated extent of drug exposure and global motion perception using multiple linear regression. This analysis was conducted only for marijuana and alcohol use, because only these two drugs had significant effects on global motion perception in the primary analysis. Furthermore, this analysis was conducted only for children who were exposed to marijuana in the absence of alcohol, or vice versa, as the effects of marijuana and alcohol were found to interact in the primary analysis.

Drug exposure was estimated from the Substance Use Inventory based on the subset of questions that addressed the frequency (how many times marijuana or alcohol was used per week) and amount (amount of marijuana or alcohol that was consumed on each occasion) of drug use. The frequency of use was categorized as <1 day per week, 1–4 days per week, or 5–7 days per week. For marijuana, the amount of drug was categorized as light (<1 joint per occasion), moderate (1–2 joints per occasion), or heavy (>2 joints per occasion). Because joints can be shared, these responses were modified to reflect the consumption of whole joints based on additional questionnaire data relating to joint sharing. For alcohol, the amount of drug was categorized as light (<2 drink per occasion), moderate (2–5 drinks per occasion), or heavy (>5 drinks per occasion). Within the Substance Use Inventory, these questions were completed separately for each trimester of pregnancy. A single estimate of frequency and a single estimate of amount of drug use were calculated for each participant from the responses for each trimester. To achieve this, we ranked the frequency of drug use in each of the trimesters (1 for <1 day a week, 2 for 1–4 days a week, and 3 for 5–7 days a week) and then took the median of the ranks across all trimesters. Similarly, the amount of drug (marijuana or alcohol) use was also ranked for each of the trimesters (1 for light users, 2 for moderate users and 3 for heavy users), and the median of the ranks across all trimesters was used for the analysis. These categories were dummy coded (no = 0, yes = 1) for multiple linear regression analyses. The multiple regression model controlled for other drug use (yes/no for methamphetamine and nicotine), sex, ethnicity, stereoacuity, visual acuity, and verbal IQ.

## Results

One hundred and seventy 4.5-year-old (54 ± 2 months) children were recruited from the New Zealand arm of IDEAL study. One hundred and sixty-five children (93 male) successfully completed a psychophysical measure of global motion perception and a comprehensive vision screening including habitual visual acuity, stereoacuity, ocular motility assessment and external eye examination. Demographically the cohort was 52.5% European, 36.5% Māori and 11% other. The children had been exposed to a range of different drugs: 75.2% to nicotine, 56.4% to alcohol, 44.2% to methamphetamine and 40% to marijuana. The majority of children (81.3%) had been exposed to multiple drugs. Twenty-five children (15%) had no drug exposure and provided a non-drug exposed comparison group.

**Figure 1:**
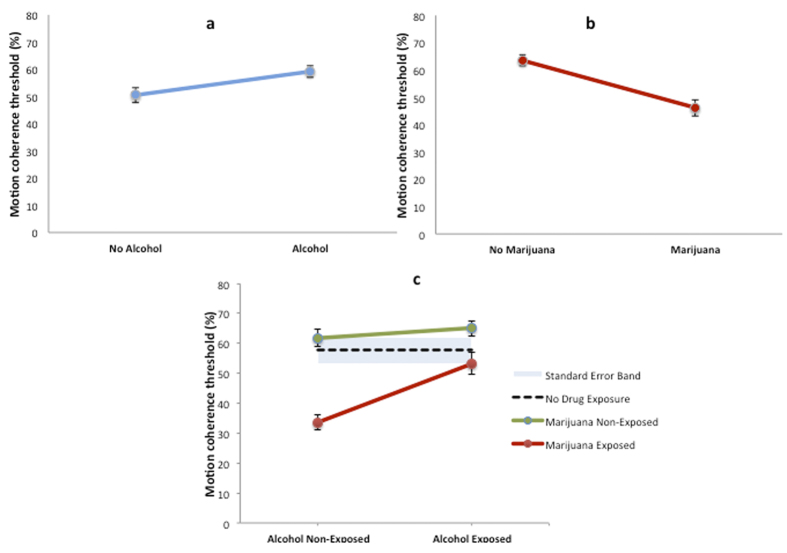
The effect of prenatal exposure to alcohol, marijuana and their combination on motion coherence thresholds, a measure of global motion perception. (**a**) Prenatal alcohol exposure (n = 95) impaired global motion perception (**b**) Prenatal marijuana exposure (n = 67) improved global motion perception (**c**) Exposure to marijuana in the absence of alcohol (n = 20) was associated with a substantial improvement in global motion perception that was significantly better (p < 0.001) than children with no history of drug exposure (dotted line, n = 25). Error bars and the shaded area show standard error of the mean.

Only alcohol and marijuana exposure had independent effects on global motion perception after controlling for the effects of multiple drug exposure, verbal IQ, ethnicity, habitual visual acuity, stereoacuity, and sex. Children who were exposed prenatally to alcohol had elevated (worse) motion coherence thresholds (Fig. 1A) whereby global motion perception was significantly poorer (t_163_ = −2.26, p = 0.002) than that of children not exposed to alcohol (alcohol exposed, n = 95: 59 ± 21% motion coherence threshold vs alcohol non-exposed, n = 70: 50 ± 23%). Unexpectedly, children exposed to marijuana had significantly lower (better) motion coherence thresholds (Fig. 1B) (t_163_ = 3.52, p = 0.001) than those of children not exposed to marijuana [marijuana exposed, n = 67: 46 ± 20% vs marijuana non-exposed, n = 98: 63 ± 25%]. A significant interaction between the effects of alcohol exposure and marijuana exposure on motion coherence thresholds was also present (Fig. 1C, F_1,114_ = 7.7, p = 0.006), whereby exposure to marijuana in the absence of alcohol was associated with improved global motion perception (mean motion coherence threshold = 34 ± 11%, n = 20), which was significantly lower (better) (t_41_ = 4.42, p < 0.001) than that of children who had not experienced prenatal drug exposure (mean motion coherence threshold = 58 ± 23%, n = 25). However global motion perception for children exposed to both marijuana and alcohol (mean motion coherence threshold = 53 ± 24%, n = 48) was no different (t_70_ = −0.28, p = 0.39) from that of children who had no drug exposure (mean motion coherence threshold = 58 ± 23%, n = 25).

No other significant main effects or interactions between drugs were found for global motion perception. Also, visual acuity (measured with habitual refractive correction if worn), stereoacuity, and verbal IQ (measured with Wechsler Preschool and Primary Scale of Intelligence-III) were unaffected by drug exposure in this group of children.

**Figure 2:**
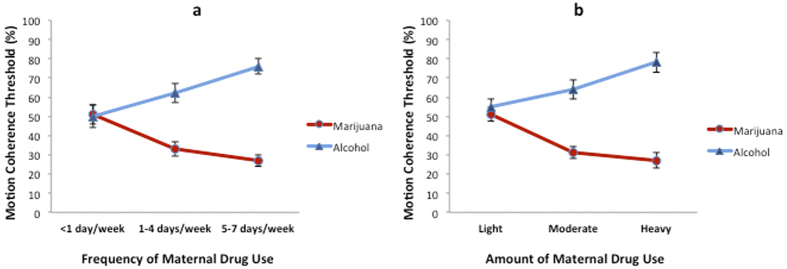
The relationship between motion coherence thresholds and the (a) frequency and (b) amount of maternal marijuana or alcohol use. The red and blue lines indicate maternal marijuana and alcohol use, respectively. Data points in panel (**a**) indicate mean motion coherence thresholds for children who were prenatally exposed to marijuana or alcohol <1 day/week (marijuana n = 6, alcohol n = 10), 1–4 days/week (marijuana n = 5, alcohol n = 14), or 5–7 days/week (marijuana n = 9, alcohol n = 22). Data points in panel (**b**) indicate mean motion coherence thresholds for children whose marijuana or alcohol exposure on each occasion was light (marijuana, <1 joint, n = 6; alcohol, <2 drinks, n = 19), moderate (marijuana, 1–2 joints, n = 7; alcohol, 2–5 drinks, n = 9), or heavy (marijuana, >2 joints, n = 7; alcohol, >5 drinks, n = 18). Drug use data are maternal self-report; see methods for further details. The error bars indicate standard error of the mean. Other drug use (yes/no for methamphetamine and nicotine), sex, ethnicity, stereoacuity, visual acuity, and verbal IQ were controlled for in the multiple regression model.

Multiple linear regression conducted on the subset of children (n = 20) who were exposed to marijuana, but not to alcohol, revealed that both the frequency of maternal marijuana use (β = −0.90; F_3,16_ = 28.19, p < 0.001; adjusted R^2^ = 0.75) and amount of joints consumed per occasion (β = −0.89; F_3,16_ = 33.26, p < 0.001; adjusted R^2^ = 0.78) had a negative linear association with motion coherence threshold. More frequent maternal use of marijuana (Fig. 2a) during pregnancy and more joints smoked per occasion (Fig. 2b) were associated with lower motion coherence thresholds, indicating better global motion perception. The opposite of this was true for children (n = 46) exposed to alcohol, but not to marijuana, whereby both frequency of maternal alcohol use (β = 0.62; F_3,42_ = 10.81, p < 0.001; adjusted R^2^ = 0.39) and amount of alcohol consumed per occasion (β = 0.67; F_3,42_ = 12.06, p < 0.001; adjusted R^2^ = 0.42) had a positive linear association with motion coherence threshold. More frequent maternal use of alcohol (Fig. 2a) during pregnancy and a greater number of alcoholic drinks per occasion (Fig. 2b) were associated with higher motion coherence thresholds, indicating poorer global motion perception.

## Discussion

Our results indicate that 1) global motion perception, a behavioural measure of extrastriate visual function, is susceptible to the effects of prenatal drug exposure, and 2) interaction effects occur when children are exposed to multiple drugs. Specifically, we found that alcohol exposure impaired, and marijuana exposure improved, global motion perception. These effects were dependant on the frequency and amount of prenatal marijuana or alcohol exposure. Furthermore, these effects appeared to cancel one another whereby prenatal exposure to both drugs resulted in no effect on global motion perception.

Our finding that prenatal alcohol exposure was associated with impaired global motion perception is consistent with previous reports documenting detrimental effects of alcohol exposure on neurodevelopment^35^ and ocular development^36^,^37^. However, the majority of prior studies have focused on children with foetal alcohol syndrome. In contrast, none of the children in this study had been diagnosed with foetal alcohol syndrome. This suggests that prenatal exposure to levels of alcohol that are not sufficient to induce foetal alcohol syndrome can still impair the cortical processing of visual information as assessed using a behavioural measure of global motion perception. Furthermore, this deficit would not be evident from the results of a vision screening, as clinical measures of visual perception such as habitual visual acuity and stereoacuity were unaffected in our group of children. However, clinical tests of stereopsis such as the VAO Fly Stereotest used in this study are unlikely to be sensitive enough to detect small differences in stereoacuity.

The finding that prenatal marijuana exposure influenced global motion perception was consistent with the idea that extrastriate visual cortex development is effected by maternal drug use. However the direction and size of the effect was unexpected; the children exposed to marijuana in the absence of alcohol were almost 50% better at the global motion task than children with no drug exposure. It is important to note that although prenatal marijuana exposure has not been studied widely, detrimental effects have been reported for motor and cognitive development^38^. Therefore our results cannot be extrapolated beyond global motion perception or interpreted as marijuana having beneficial effects on foetal development. Furthermore, superior performance on a single behavioural task does not necessarily indicate supernormal neurodevelopment. For example, children with autism have superior performance on a mirror symmetric global pattern task compared to controls^39^; however their motor control is poorer than non-autistic individuals^40^,^41^,^42^.

One possible explanation for the improved global motion perception we observed in marijuana-exposed children relates to the potential neurochemical effects of this drug on the developing visual system. Endocannabinoid receptors are present throughout the visual pathway of non-human primates and have particularly high expression within dorsal stream brain areas such as the middle temporal area (MT; the primate equivalent of human V5) and the middle superior temporal area (MST)^43^. Both MT/V5 and MST are motion sensitive areas within the extrastriate visual cortex of humans and non-human primates^44^,^45^. Although the role of endocannabinoid receptors in dorsal stream function is unknown, there is evidence that cannabinoids can enhance the function of specific neural pathways. For example, cannabinoids improve movement and locomotion of rats in a dose-dependent manner^46^. Therefore, it is possible that prenatal marijuana exposure acts upon endocannabinoid receptors in MT/MST in a way that improves global motion perception in human infants. It has also been found that cannabinoids facilitate neurogenesis by acting as anti-oxidants and regulating mitochondrial activity in preclinical models of neurodegenerative disorders^47^. These effects may also occur within the extrastriate visual cortex.

Previous studies have reported that prenatal exposure to heavy marijuana use impairs performance on a range of standardized neuropsychological tests of attention, memory, and executive function that involve a visual component^48^,^49^,^50^,^51^,^52^,^53^. These tests typically require the encoding, memorization, and recognition of visual patterns and objects. Therefore, they may involve processing within the ventral visual stream that includes regions of extrastriate visual cortex specialized for object recognition^26^,^27^. In combination with our results, this raises the interesting possibility that prenatal marijuana exposure may improve dorsal stream function but impair ventral stream function. Alternatively, the detrimental effects of marijuana on visual processing reported in previous studies may reflect impairments at the level of visual attention, visual memory, or response inhibition rather than visual perception^53^.

The antagonistic effects we report for marijuana and alcohol exposure on motion processing highlight the importance of considering poly-drug interactions when investigating the consequences of prenatal drug exposure. Marijuana neutralizes the effect of nicotine in an animal model of addiction^54^; however interactions between drugs administered prenatally have not been explored comprehensively in animal models. If antagonistic drug effects on foetal and infant neurodevelopment can be confirmed, a pathway for the development of interventions that minimize the harmful effects of prenatal drug exposure may be opened.

The absence of any effect of prenatal nicotine exposure on global motion perception is consistent with previous neuropsychological studies suggesting that nicotine impairs global cognitive function and auditory processing rather than tasks that involve visual processing^49^,^48^. However, deficits in visual attention have been reported in children with prenatal nicotine exposure^55^. Our data indicate that any deficits in visual attention do not impact on habitual visual acuity, stereopsis, or global motion perception in children with prenatal nicotine exposure. Similarly, prenatal exposure to methamphetamine did not affect habitual visual acuity, stereopsis, or global motion perception in our study cohort. No previous prospective studies have investigated the effect of methamphetamine on visual development in humans, although there is evidence for ocular and optic nerve abnormalities in rats parentally exposed to methamphetamine^56^,^57^. No children in our study exhibited visual deficits that would suggest serious ocular or optic nerve pathology; however retinal structure was not specifically investigated as part of our study protocol.

The average motion coherence thresholds we report for non-drug exposed children are slightly elevated (worse) compared to a number of previous studies of global motion perception in preschool children^58^,^59^. This was likely because of differing stimulus parameters and psychophysical techniques. However, because children within the IDEAL study were matched for factors such as socioeconomic status and maternal education, it is possible that low socioeconomic status and its related risk factors had a negative impact on global motion development for the whole group. We also note that other studies have reported higher (worse) global motion thresholds than ours for normally developing children of a similar age^60^.

Overall, our results demonstrate that the development of global motion perception is affected by prenatal exposure to alcohol or marijuana but not nicotine or methamphetamine. Our finding that global motion perception was improved by marijuana exposure and that marijuana reduced the negative effect of alcohol exposure may provide a foundation for further studies investigating new ways to prevent or ameliorate the negative developmental effects of prenatal drug exposure.

## Additional Information

**How to cite this article**: Chakraborty, A. *et al.* Prenatal exposure to recreational drugs affects global motion perception in preschool children. *Sci. Rep.* **5**, 16921; doi: 10.1038/srep16921 (2015).
